# Risk Awareness, Self-Efficacy, and Social Support Predict Secure Smartphone Usage

**DOI:** 10.3389/fpsyg.2020.01066

**Published:** 2020-06-12

**Authors:** Guangyu Zhou, Mengke Gou, Yiqun Gan, Ralf Schwarzer

**Affiliations:** ^1^Beijing Key Laboratory of Behavior and Mental Health, School of Psychological and Cognitive Sciences, Peking University, Beijing, China; ^2^Department of Education and Psychology, Health Psychology, Freie Universität Berlin, Berlin, Germany; ^3^SWPS University of Social Sciences and Humanities, Wrocław, Poland

**Keywords:** self-efficacy, risk awareness, social support, smartphone security behaviors, information security

## Abstract

It is widely acknowledged that non-compliance with smartphone security behaviors is widespread and may cause severe harm to people and devices. In addition to device-based security issues, there are psychological factors involved in these behaviors such as self-efficacy, risk awareness, and social support. The present study examines associations of these three factors with smartphone security behaviors and explores possible mechanisms among these variables. In a longitudinal survey with 192 Chinese college students (73.4% women, mean age 24.46 years, SD = 5.15), self-efficacy, risk awareness, and social support were assessed with psychometric scales at two points in time, 2 weeks apart. Hierarchical regression analyses were performed with follow-up smartphone security behaviors as the dependent variable, controlling for baseline values and demographic and IT-related covariates. Main effects of self-efficacy, risk awareness, and social support on smartphone security behaviors were identified. Moreover, a triple interaction among the three predictors emerged in a synergistic way, indicating that their combination yielded more favorable levels of secure smartphone use. The total model accounted for 50% of the behavioral variance, with all covariates included, and the triple interaction among self-efficacy, risk awareness, and social support accounted for 2.3% of variance. Results document that psychological factors are involved in smartphone security behaviors beyond demographic and IT-related covariates. Interventions could be designed to improve smartphone security behaviors not only by developing privacy-enhancing technologies but also by considering psychological factors such as self-efficacy, risk awareness, and social support.

## Introduction

A smartphone is a multifunctional mobile device with a particular operating system, which could have high-speed Internet access, and mobile applications in addition to basic features of a traditional telephone (Wang et al., 2014; Hossain and Ahmed, 2016). The number of smartphone users has increased rapidly in the past decade. By the end of 2018, there have been 3 billion smartphone users worldwide and 783 million of them in China (Emma, 2018). Along with the extensive smartphone usage, a growing number of security issues have surfaced, especially for malicious interception of personal information, which led to data hacking, and property damage (Zhang et al., 2017). Smartphone malware attacks are consistently reported in forms of viruses, worms, Trojan horses, phishing messages, and spyware (Leavitt, 2005; Wang et al., 2012; Jones and Chin, 2015), which could result not only in impaired services and poor mobile phone performance but also in jeopardized resources (Wang et al., 2012).

Numerous prior studies have claimed the significance of complying with smartphone security behaviors to protect information security (Jones and Heinrichs, 2012; Wang et al., 2012; Das and Khan, 2016; van Schaik et al., 2018). Various available security behaviors have been strongly recommended by computer professionals. These measures have included installing antivirus applications (Jones and Heinrichs, 2012), taking precautions through phone settings such as passwords, remote services, data backup, and personal blacklists (Das and Khan, 2016; Zhang et al., 2017), as well as avoiding harmful or suspicious web links, Wi-Fi, and Bluetooth transmissions (Wang et al., 2012). A study identified 28 cybersecurity behaviors and practices, which smartphone users should follow to improve cybersecurity (Shah and Agarwal, 2020). In reality, many smartphone consumers do not adopt appropriate security behaviors in spite of their knowledge of smartphone security threats.

A great number of studies have concentrated on the determinants of online information security with computer use. Although security laws and organizational policies have been widely used to prevent the occurrence of security risk–taking and damaging behaviors in companies and work situations (Guo, 2013), the role of personal factors remains unclear. Other than policy-impelled contexts, this study explores self-directed smartphone security behavior and its possible mechanisms in everyday usage. Among personal factors are social-cognitive constructs such as risk awareness, social support, and self-efficacy. At the individual level, self-efficacy, and appraisal of potential threats are considered to impact the adoption of secure computer usage. Self-efficacy pertains to one’s optimistic belief in being able to overcome barriers or meet challenges while pursuing a goal. When it comes to taking security precautions, self-efficacy refers to one’s belief in being competent to employ appropriate measures to counteract a smartphone security risk. Research has shown that self-efficacy was associated with an Internet consumer’s intention to adopt secure behaviors (Ng et al., 2009). Response self-efficacy, which was interpreted as a measure of one’s perceived resources, tended to boost precautionary online behaviors (Jansen and Van Schaik, 2017), and to reduce the probability of information security behavior lapses (Workman et al., 2008).

Another prerequisite for taking precautions is the awareness of risk. Research has shown that threat appraisal played an important role in determining security behavior. Perceived susceptibility of a security threat and perceived benefit of a security behavior were associated with decisions about the handling of email attachments (Ng et al., 2009). Consistently, perceived susceptibility and severity of security threatening factors had positive associations with secure smartphone usage (Verkijika, 2018).

In addition to self-efficacy and risk awareness, social support from family members, friends, or co-workers might be facilitators for secure smartphone usage. Although there is no direct evidence on the relationship between social support and information security behaviors, some studies give a hint in this direction. For instance, an employee’s involvement in a group might motivate help-seeking behaviors when being confronted with an IT security dilemma (Dang-Pham et al., 2019). Another study found that smartphone users’ behavioral intent of applying anti-spy services was associated with social contexts that were supportive to implementing secure measures (Bulgurcu et al., 2010; Johnston and Warkentin, 2010). To further examine the role of social support in secure smartphone usage, empirical research with social support as a predictor is needed.

### Aims

The current study aims at examining social-cognitive antecedents involved in secure smartphone usage, namely risk awareness, self-efficacy, and social support. We expect positive associations (main effects) of all three factors on secure smartphone use. Security behavior requires some level of risk awareness; otherwise, one is hardly motivated to make a change. Also, self-efficacy, i.e., feeling competent to execute such action, needs to be present to some degree; otherwise, people would not take initiative. Moreover, social support constitutes a facilitating factor, making it easier to implement protective measures. In addition to these hypothesized three main effects, we will examine interactions among the predictors to uncover patterns that may shed light on possible mechanisms in the security behavior adoption process.

## Materials and Methods

### Participants

College students from a university located in Beijing, China, were invited to attend a two-wave survey during class breaks. A total of 192 students gave their consent and completed a battery of questionnaires at baseline (Time 1) regarding Internet and smartphone usage. There were 173 (90.10%) of them retained to report their smartphone security behavior after 2 weeks (Time 2). Psychosocial variables related to smartphone security use including self-efficacy, risk awareness, and social support were assessed at Time 1, as well as demographic information. Smartphone security behavior was assessed at both time points. Approximately 3 United States dollars (20 RMB) was given to participants who completed the whole study as appreciation of their efforts. The study protocol has been approved by the Human Research Ethics Committee at the School of Psychological and Cognitive Sciences of Peking University.

### Measures

Measurement of smartphone security use behavior was adapted from Zhang et al. (2017) scale on information security behaviors of smartphone users in China. For simplicity, one item from avoidance of harmful behaviors and two items from use of phone settings and add-on utilities were used in the current study. A similar scale has been adapted in India (Shah and Agarwal, 2020). Social-cognitive variables including self-efficacy, risk awareness, and social support were assessed with tools adapted from previous research concerning health behaviors such as dietary behavior and facemask wearing (Zhou et al., 2015, 2016). Based on previous empirical studies inspired by the health action process approach (HAPA; Schwarzer, 2008), we modified at least five items to tap each social-cognitive variable to the context of smartphone security usage. All measures are provided in the section Appendix.

#### Self-Efficacy

Self-efficacy of smartphone security use pertained to the perception of an individual’s capability to take precautions, for example, to lower hacker attacks in daily smartphone usage. Self-efficacy was assessed with four items at Time 1. One sample item was “It’s easy for me to adhere to sound and secure behaviors when using my smartphone.” Responses were rated on a five-point scale from 1 (completely disagree) to 5 (completely agree). A total score was calculated by summing up the four items. A higher sum score indicated higher self-efficacy to use the smartphone properly. Cronbach’s α in the current sample was.88.

#### Risk Awareness

Risk awareness referred to one’s perception of how harmful it could be in the case of not using the smartphone securely. It was measured with three items at Time 1. One sample item was “If I don’t take any security precaution in using my smartphone, my private information will be hacked.” Each item was rated on a five-point scale from 1 (completely disagree) to 5 (completely agree). The three items were summed up to a total score. A higher sum score indicated higher risk awareness. Cronbach’s α of the scale in the current sample was.73.

#### Social Support

Three items were adopted to assess received social support at Time 1 regarding smartphone security use during the past week. An example item was “My friends or family members reminded me to take specific precautious measures in smartphone usage.” Items were rated on a five-point scale with 1 = completely disagree and 5 = completely agree. The three items were summed up to a total score. A higher sum score indicated a higher level of social support. Cronbach’s α of the social support scale in the current study was.87.

#### Secure Smartphone Usage

Secure smartphone usage was assessed with a behavioral index at Time 1 and Time 2. The behavioral index was composed of three items to assess smartphone behaviors in different aspects in the past week. These aspects included the updating of antivirus programs, security evaluation before installing an application, and disabling Global Position System (GPS)/Bluetooth while not in use. A sample item was “I verified its source and checked its evaluation before installing a new App.” All items were rated from 1 = completely disagree to 5 = completely agree. A total score was calculated by summing up the three items. A higher score indicated safer smartphone usage.

#### Potential Covariates

Potential covariates including demographic information, data plan purchase, hacker attack experience, experience of property damage due to smartphone security vulnerabilities, smartphone use experience, Internet use experience, and operating system of the smartphone were assessed. To be more specific, data plan purchase, hacker attack experience, and experience of property damage due to smartphone security vulnerabilities were each measured with a dichotomous item. To control for the experience of using smartphones and the Internet, the following question was included: “How many years have you been using a smartphone/the Internet?”

### Data Analysis

First, descriptive analyses for baseline and follow-up samples were performed. Second, an attrition analysis was conducted to examine the difference between participants who dropped out and those who were retained. Third, bivariate correlations were calculated among the main variables. Finally, hierarchical regression analyses were carried out with self-efficacy, risk awareness, social support, and a triple interaction of three social-cognitive variables as independent variables, and smartphone security behavior as the dependent variable. The estimates of coefficients and bootstrapped 95% confidence intervals (CIs) were based on 5,000 resamples. A *p*-value less than.05 or a CI not including 0 was the indicator of statistical significance. There were less than 7% missing values, and Little’s Missing Completely at Random (MCAR) test was not significant. Missing data were imputed by the expectation maximization (EM) procedure. All analyses were performed with SPSS 25 and the SPSS PROCESS macro (Hayes, 2013).

## Results

### Descriptive Statistics

Of the study population, 141 participants (73.40%) were women, with a mean age of 24.46 years (SD = 5.15). Half of the participants (*n* = 98, 51%) majored in humanities and social sciences. There were 20 participants (10.40%) who majored in information security. The majority of participants (*n* = 173, 90.10%) purchased a data plan. A small number of participants had been hacked (*n* = 13, 6.80%) or experienced damage to properties due to insecure smartphone use (*n* = 12, 6.30%). The average time span of smartphone and Internet use were 5.83 years (SD = 2.50) and 10.92 years (SD = 3.62), respectively. Their smartphone operating system included Android (*n* = 49, 25.50%), iOS (*n* = 81, 42.20%), and Windows (*n* = 61, 31.80%). Detailed descriptive statistics are shown in Table 1.

**TABLE 1:** Descriptive statistics of participants, social-cognitive variables, and behaviors.

	*M*/*N ^a^*	SD/%^a^	Range	Missing (%)
Age (years)	24.48	5.30	18–47	6.30
Gender (female)	141	73.40	–	3.10
Major				4.20
Science and technology	39	20.30	–	
Humanities and social sciences	98	51.00	–	
Medicine	47	24.50	–	
Major in information security (yes)	20	10.40	–	0
Data plan purchase (yes)	173	90.10	–	0
Hacked experience (yes)	13	6.80	–	0
Property damage due to smartphone	12	6.30	–	0
insecurity use (yes)				
Smartphone use experience (years)	5.83	2.50	2–13	0
Internet use experience (years)	10.92	3.62	0–22	0
Smartphone operating system				0.50
Android	49	25.50	–	
iOS	81	42.20	–	
Windows	61	31.80	–	
Secure smartphone usage at Time 1	9.85	2.54	3–15	0.50
Risk awareness at Time 1	11.17	2.38	3–15	0
Self-efficacy at Time 1	13.54	3.27	4–20	0.50
Social support at Time 1	7.71	3.28	3–15	0.50
Secure smartphone usage at Time 2	10.53	2.58	3–15	9.90

*^*a*^For categorical variables.*

### Attrition Analysis

At Time 2, 19 (9.9%) participants dropped out from the study. Attrition analysis reveals that there were no significant differences between participants who completed the study and those who dropped out in terms of most variables measured at Time 1. For exceptions, participants who completed the survey were significantly older (*t* = 4.49, *p* < 0.01), and less likely to be majoring in science and technology (χ2 = 12.93, *p* < 0.01) than those who dropped out. Moreover, participants who completed the study reported better baseline values in smartphone security behavior (*t* = 2.01, *p* < 0.05).

### Associations Among Key Variables

There was a significant association between secure smartphone usage at Time 1 and Time 2 (*r* = 0.57, *p* < 0.01). Social-cognitive variables at Time 1, including self-efficacy (*r* = 0.32, *p* < 0.01), risk awareness (*r* = 0.39, *p* < 0.01), and social support (*r* = 0.43, *p* < 0.01), were positively correlated with secure smartphone usage at Time 2. No gender differences emerged. Older individuals had more smartphone and Internet experience than younger ones, and they reported a higher frequency of property damage and higher levels of social support (see Table 2).

**TABLE 2:** Correlation matrix of main variables (*N* = 173).

	1	2	3	4	5	6	7	8	9	10	11
Gender											
Age	−0.15*										
Data plan purchase	0.10	–0.02									
Hacked experience	−0.15*	0.14	–0.05								
Property damage	–0.04	0.19**	–0.06	0.27**							
Smartphone use experience	–0.09	0.29**	–0.06	0.12	0.04						
Internet use experience	–0.03	0.27**	0.12	0.01	–0.11	0.36**					
Secure smartphone usage at Time 1	–0.05	0.05	–0.13	0.04	–0.04	–0.001	–0.06				
Risk awareness at Time 1	–0.09	–0.08	–0.05	0.15*	0.07	–0.12	–0.10	0.26**			
Self-efficacy at Time 1	0.10	–0.05	–0.11	0.060	–0.08	0.07	0.14	0.22**	0.07		
Social support at Time 1	–0.10	0.21**	–0.14	0.06	0.07	0.11	0.03	0.36**	0.26**	0.25**	
Secure smartphone usage at Time 2	0.02	0.04	–0.09	0.14	0.08	0.06	–0.10	0.57**	0.40**	0.34**	0.43**

*p < 0.05; **p < 0.01.

### The Joint Associations of Social-Cognitive Predictors With Secure Smartphone Usage

A hierarchical regression analysis was performed to examine the effects of psychosocial determinants of secure smartphone usage. In the first step, baseline variables including gender, age, data plan purchase, hacked experience, property damage, smartphone use experience, Internet use experience, and secure smartphone usage were regressed on smartphone security usage at Time 2, accounting for 35% of the behavioral variance (*p* < 0.001). In the second step, self-efficacy, risk awareness, and social support at Time 1 were added as independent variables, accounting for an additional 14% of behavior variance (*p* < 0.001). In the third step, a triple interaction of social-cognitive variables was added, accounting for 2.3% of additional variance (Δ*R*^2^ = 0.023, *p* = 0.005). The regression model totally explained 50% of the variance in behavior at Time 2 with all covariates included. Without covariates, the model with three predictors accounted for 33% of the variance. Results of the hierarchical model are displayed in Table 3.

**TABLE 3:** Secure smartphone usage at Time 2 regressed on self-efficacy, risk awareness, social support, and a triple interaction of the social-cognitive variables at Time 1, controlling for age, sex, smartphone experience, and baseline behavior.

	Coefficients			Model summary	
	*B* (95% CI)	β	*t*	*R*^2^	*p*
Model 1^a^				0.35	<0.001
Model 2^b^				0.48	<0.001
Model 3^c^				0.50	0.005
Gender	0.47(−0.18,1.11)	0.08	1.42		
Age	−0.01(−0.06,0.05)	–0.01	–0.16		
Data plan purchase	0.40(−0.53,1.33)	0.05	0.85		
Hacker experience	0.44(−0.72,1.59)	0.04	0.75		
Property damage	0.59(−0.62,1.80)	0.06	0.96		
Smartphone use experience	0.14(0.01,0.26)	0.13	2.16*		
Internet use experience	−0.10(−0.19,−0.02)	–0.15	−2.40*		
Secure smartphone usage at Time 1	0.41(0.29,0.53)	0.41	6.97**		
Risk awareness at Time 1	0.26(0.14,0.38)	0.24	4.21**		
Self-efficacy at Time 1	0.19(0.10,0.28)	0.25	4.21**		
Social support at Time 1	0.14(0.04,0.23)	0.18	2.86**		
Risk*Self*Social at Time 1	−0.35(−0.59,−0.11)	–	−2.87**		

*B* = unstandardized regression coefficient, β = standardized regression coefficient, *p < 0.05, **p < 0.01. ^*a*^Gender, age, data plan purchase, hacked experience, property damage, smartphone use experience, Internet use, and secure smartphone usage at Time 1 are included as independent variables in Model 1. ^*b*^Self-efficacy, risk awareness, and social support at Time 1 are additionally included in Model 2. ^*c*^A triple interaction among the psychosocial variables is included in Model 3.

**FIGURE 1:**
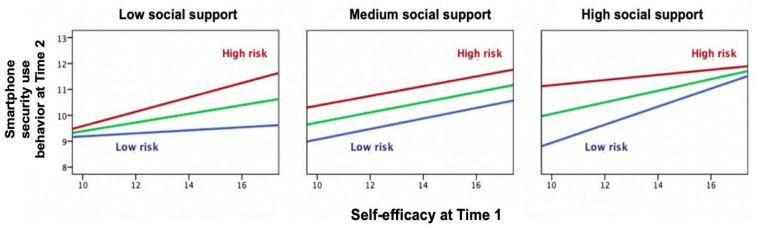
Triple interaction among self-efficacy, risk awareness, and social support in secure smartphone usage at Time 2, controlling for sex, age, data plan purchase, hacked experience, property damage, smartphone use experience, Internet use experience, and secure smartphone usage at Time 1.

Figure 1 displays the pattern of the triple interaction among three social-cognitive variables in predicting secure smartphone usage. High self-efficacy, high risk awareness, and high social support were associated with more secure smartphone usage. Lack of self-efficacy and risk awareness indicated the lowest level of secure smartphone usage in all three panels. Moreover, the patterns of simple slopes differed among levels of independent variables. In the leftmost panel, in the subgroup of participants with lower support, there was an ordinal interaction between self-efficacy and risk awareness in a synergistic manner, meaning that the combination of both predictors yielded the highest level of secure smartphone usage, although it was lower than in the other panels. The variance was highest in individuals with high self-efficacy, whereas in the rightmost panel, the variance was highest in those with low self-efficacy. In this high-support group, the most secure smartphone usage was found, but only when self-efficacy was also high.

## Discussion

This study focused on social-cognitive determinants of secure smartphone usage within a longitudinal research design. Results indicated that self-efficacy, risk awareness, and social support significantly predicted smartphone security behavior. Moreover, a triple interaction of the three social-cognitive variables on secure smartphone usage emerged. Different levels of secure smartphone behavior appeared in various patterns of self-efficacy, risk awareness, and social support.

The main finding of this study was the direct effects of self-efficacy, risk awareness, and social support on secure smartphone usage. The overall regression model revealed a positive prediction of self-efficacy, risk awareness, and social support at Time 1 for the prediction of Time 2 smartphone security behavior, as well as an explanation of 50% of the variance of Time 2 smartphone security behavior including covariates, 33% without covariates. Security behavior seems to benefit from a certain level of risk awareness because without any awareness, people are hardly motivated to make a change in their daily smartphone use. Moreover, feeling competent to execute preventive actions (self-efficacy) facilitates one’s motivation to take initiative. Being embedded in a social network that provides information and tangible support makes it easier to implement protective measures.

The findings are consistent with previous work that reported positive associations between self-efficacy and secure behavior (Workman et al., 2008; Das and Khan, 2016; Jansen and Van Schaik, 2017). Also, risk awareness and perceived threat were associated with smartphone security behaviors (Ng et al., 2009) and, in addition, interactions and joint effects of these two factors (Fenz et al., 2014; Das and Khan, 2016). In the interpersonal context, social support from family, friends, and colleagues was related to a higher level of individuals’ smartphone security behavior. This finding could be regarded as an implication of social norms and the organizational socialization in companies, resulting in the collective adoption of smartphone security behaviors (Johnston and Warkentin, 2010; Alsaleh et al., 2017; McGill and Thompson, 2017; Dang-Pham et al., 2019). Due to social support by close families and friends and particular recommendations of adopting smartphone security behaviors, a feeling of immersion and having the concerned of others may explain the elevated smartphone security behavior. This, way, social support could trigger normative beliefs about smartphone security use behaviors.

The subsequently identified triple interaction qualified the main effects in a synergistic manner because a combination of the single associations resulted in a higher level of secure smartphone use. This finding was a validation of determining effects of self-efficacy, threat severity, and social influence in human–computer interactions (Johnston and Warkentin, 2010). In spite of the low amount of explained variance, there was still a clear tendency for the joint effects of self-efficacy and risk awareness to vary from low to high social support levels, as displayed in Figure 1, with a divergent pattern of low social support and a convergent one of high social support. In general, people with higher risk awareness (Albrechtsen and Hovden, 2010), or self-efficacy (Arachchilage and Love, 2014) obtain a higher level of smartphone security use. The slope for low risk awareness became steeper when medium or high social support was reported, which may reflect a compensation of interpersonal support for personal deficits. In the situation of medium social support, the interaction between self-efficacy and risk awareness disappeared. In consideration of practical implications, the promotion of smartphone security behavior should highlight the importance of risk awareness for users with high self-efficacy and the beneficial effect of social support for users with low risk awareness.

There are several limitations of this study that should be addressed in future research. First, the sample consisted of Chinese students with a mean age of 24.46 years (SD = 5.15), and there may be different effects in other-generation samples. Second, all data were self-reported, and it would be superior to add objective data on smartphone security behaviors. However, recording or observing such objective data requires a complicated and resource-demanding research design. Third, the reliability coefficients of the adapted scales were a bit lower than ideal, and one would like to obtain more advanced measurement tools. Further, this was a longitudinal research design that does not allow for causal inferences. Moving forward to design a randomized controlled trial would be an advantage.

Nevertheless, the present data may stimulate further research on the psychosocial mechanisms that are involved in the adoption and maintenance of smartphone security behaviors. Including self-efficacy, risk awareness, and social support in such research appears to be valuable. Moreover, when it comes to intervention designs, one could point out to customers the objective levels of security risks along with instilling optimistic self-beliefs on how to cope with such challenges. Mobilizing and providing social support to prevent a security risk or to adopt precautions would be a good idea. Developing privacy-enhancing technologies would facilitate the personal efforts to implement secure smartphone use.

## Data Availability Statement

The datasets generated for this study are available on request to the corresponding author.

## Ethics Statement

The studies involving human participants were reviewed and approved by the Institutional Review Board at Peking University School of Psychological and Cognitive Sciences. The patients/participants provided their written informed consent to participate in this study.

## Author Contributions

GZ, YG, and RS constructed the study protocol. GZ secured the grant. MG analyzed the data and initially drafted the manuscript. RS also made great contributions to editions and valuable comments.

## Conflict of Interest

The authors declare that the research was conducted in the absence of any commercial or financial relationships that could be construed as a potential conflict of interest.

## Appendix

**English translation of scales** (item responses range from 1 = completely disagree to 5 = completely agree):

### Smartphone Security Behavior

In the past month, what did you do to make sure that your smartphone was safe?

1. I frequently updated the operating system and antivirus software in my phone.
2. Before installing an App, I always read the comments to find out if it’s safe.
3. I always turn off GPS/Bluetooth when it’s not in use.

In the following statements, “smartphone security behavior” mainly refers to the timely update of the system and anti-virus software, not downloading and installing potential risky applications (Apps), and timely shutdown of GPS/Bluetooth.

### Risk Perception

What harm do you think it will do if you don’t practice smartphone security behaviors?

If I don’t take safety precautions, then.

1. My phone will run slowly.
2. My privacy will be at risk.
3. My money will be lost.

### Self-Efficacy

To what extent do you believe you can successfully adopt smartphone security behavior?

1. I have the necessary knowledge and skills to protect my mobile phone.
2. Adopting smartphone protection is easy for me.
3. I believe I am capable of protecting my phone from hackers.
4. Adopting smartphone protection is under my control.

### Social Support

To what extent do your friends or family support your adoption of mobile security behavior?

In the last month, my friends or family.

1. Reminded me to take smartphone security precautions.
2. Helped me with my smartphone security behavior.
3. Joined me in adopting smartphone security behavior.
